# Hedonic contrast under misleading labels: Bidirectional effects across sweet and salty foods

**DOI:** 10.1016/j.crfs.2026.101420

**Published:** 2026-05-01

**Authors:** Norbert Vanek, Ziyi Zhuang, Danaé Larsen

**Affiliations:** a Sensory Psycholinguistics Lab, Faculty of Arts and Education, University of Auckland, 18 Symonds Street, Auckland, 1010, New Zealand; b Experimental Research on Central European Languages Lab, Charles University, Jana Palacha 1/2, 116 38 Prague 1, Prague, 11000, Czechia; c School of Chemical Sciences, Faculty of Science, University of Auckland, 23 Symonds Street, Auckland, 1010, New Zealand

**Keywords:** Expectancy violation, Food labelling, Hedonic contrast, Flavour perception, Sensory reweighting

## Abstract

Misleading food labels generate powerful expectations that shape both anticipatory sensory judgments and hedonic evaluation, as demonstrated in the classic smoked salmon ice-cream paradigm (Yeomans et al., 2008, FQP). The present study replicates the core effect and broadens the paradigm in three specific ways. First, rather than relying on a single stimulus, we generalised the paradigm across ten food stimuli spanning two opposing flavour trajectories, five Sweet-to-Salty (*e.g. Blue-cheese smoothie, Tuna yoghurt*) and five Salty-to-Sweet (*e.g. Caramel tofu cubes, Chocolate-sauce mashed potato*). Second, we examined expectancy-violation effects bidirectionally. Third, we isolated label-based expectations by eliciting pre-taste ratings from written labels alone, without visual cues. Forty Mandarin-dominant participants evaluated all items under three label conditions (Expected, Unexpected, Neutral) in a two-phase procedure, first rating each item from its written label alone and then re-rating it after tasting. In the Sweet-to-Salty direction, misleading labels produced strong anticipatory inflation of sweetness and pleasantness, followed by sharp post-taste declines, accompanied by expectancy-sensitive increases in perceived saltiness. Robust Condition × Time interactions indicated graded re-evaluation under mismatch. In the Salty-to-Sweet direction, expectancy effects were again strongest prior to tasting, particularly for sweetness and saltiness judgments. Sensory experience substantially reduced or reversed these anticipatory divergences, especially under Unexpected labelling, although hedonic re-evaluation was less trajectory-sensitive and some residual differences remained for saltiness. Together, the findings show that linguistic labels alone can establish strong anticipatory priors across multiple food types and flavour trajectories that are then dynamically recalibrated in light of sensory input. The magnitude and symmetry of this reweighting depended on flavour direction and judgement type, indicating that expectancy-driven contrast effects are robust but dimension-specific. By extending the paradigm beyond a single food and incorporating bidirectional trajectories, the study sheds new light on how verbal labels shape flavour perception and consumer evaluation in ways that are consistent with predictive sensory reweighting.

## Highlights

• Written labels alone generated strong anticipatory priors for sweetness, saltiness, and pleasantness.
• Expectancy-violation effects generalised beyond smoked salmon ice-cream to ten unusual food items.
• Hedonic contrast was strongest when sweet expectations were violated by savoury-dominant foods.
• Sensory reweighting was bidirectional, but its expression depended on flavour trajectory and judgment type.

## Graphical abstract

**Figure:**
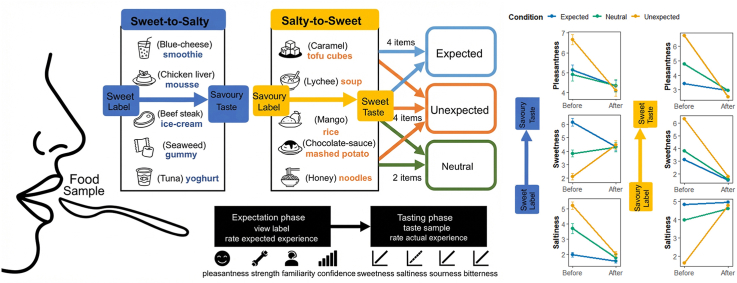

## 1 Introduction

### 1.1 Multisensory flavour perception

Flavour perception in everyday food consumption is inherently multisensory. Rather than reflecting taste alone, flavour emerges from the integration of gustatory, olfactory (ortho- and retronasal), tactile, thermal, and other sensory inputs (Auvray and Spence, 2008; Delwiche, 2004; Small et al., 1997; Qiu et al., 2025; Spence, 2015). In this sense, what consumers commonly describe as the “taste” of a food is more accurately understood as flavour, a composite sensory experience shaped by multiple channels of input rather than by gustation in isolation. This distinction is central to the present study. Throughout, we use *gustation* to refer narrowly to basic taste qualities, and *flavour* to refer to the broader multisensory percept that integrates gustatory, olfactory and trigeminal information (Auvray and Spence, 2008; Small and Prescott, 2005). Because our stimuli consist of real foods and food-like preparations that engage both taste and retronasal olfaction, the judgments examined here are more precisely judgments of perceived flavour rather than isolated taste.

Although the sensory mechanisms underlying flavour are grounded in shared neurobiology, flavour perception is highly susceptible to modulation by learning, context and prior experience (Faurion et al., 1998, 2002). Expectations generated before ingestion can bias how incoming sensory information is interpreted, weighted and evaluated. Visual properties such as colour and appearance are well-known to shape flavour identification and liking, often through learned associations acquired over repeated exposure (Dubose et al., 1980; Koster et al., 2004; Morrot et al., 2001; Stillman, 1993). More broadly, these findings show that flavour perception is not purely stimulus-driven. It reflects an interaction between bottom-up sensory evidence and top-down expectations, making multisensory flavour perception a particularly revealing domain in which to study the effects of labelling and expectancy. Recent neuroscientific approaches, including EEG-based studies of food perception (*e.g.*, Yang et al., 2025), offer promising tools for tracking the temporal dynamics of flavour processing. At the same time, such methods are sensitive to artefacts, environmental noise, and participant-related variability (Bigdely-Shamlo et al., 2015), which makes careful preprocessing and the development of more standardised data practices especially important in this emerging area of research.

### 1.2 Labelling, expectations, assimilation and contrast

Expectations also arise from verbal and written labels, which often guide food product choice and evaluation before any sensory contact occurs. A substantial body of literature demonstrates that labels and descriptions can modify both perceived sensory qualities and liking (Deliza and MacFie, 1996). In many cases, expectations produce assimilation effects, whereby actual evaluations shift toward the anticipated qualities. For example, labels implying higher quality or pleasantness tend to increase liking and enhance congruent sensory attributes such as sweetness or creaminess (Cardello and Sawyer, 1992; Wansink et al., 2005). Recent work further shows that descriptors referring to alcohol content, sensory intensity or health-related properties can systematically bias perceived flavour attributes and hedonic judgments (Blackmore et al., 2022). However, expectancy effects do not always result in assimilation. When the discrepancy between expected and actual sensory properties is large and salient, contrast effects may occur, leading to reduced liking and exaggerated perception of unexpected attributes. According to discrepancy-based models of expectation (*e.g.*, Wilson et al., 1989), small or unnoticed mismatches promote assimilation, whereas explicit violations trigger contrast. Empirical evidence for contrast effects is most robust in studies using highly novel or implausible food combinations or intentionally mislabelled products (Zellner et al., 2004), where expectations are clearly disconfirmed. For example, labelling a bitter breath freshener as *Japanese candy* led participants to rate it as even more unpleasant after tasting, whereas describing a pleasant fruit drink as *cough syrup flavouring* amplified its post-taste pleasantness, demonstrating a clear bidirectional contrast under intentional mislabelling. The present study was designed to fall on this contrast-inducing side of the discrepancy continuum. The selected foods were paired with labels that implied a strongly opposing flavour profile, namely, sweet-associated labels applied to savoury- or umami-dominant products, and savoury-associated labels applied to foods with clearly sweet tastes. Because these pairings create large and perceptually salient mismatches between the expected and experienced flavour direction, they were expected to exceed the discrepancy threshold typically associated with assimilation and instead elicit contrast.

### 1.3 Sensory vs. hedonic contrasts and the smoked salmon ice-cream paradigm

Relatively few studies have examined how strongly disconfirmed expectations simultaneously affect sensory and hedonic evaluations, particularly under conditions where the mismatch is stark. Early work using mislabelled taste solutions suggested sensory contrast effects (Carlsmith and Aronson, 1963), but these findings were often interpreted in affective terms. More recent evidence indicates that sensory attributes such as sweetness and bitterness can vary independently of liking (Yeomans et al., 2006), which highlights the need to distinguish sensory reorganisation from hedonic responses. The smoked salmon ice-cream paradigm was developed to address this distinction. By creating a highly novel food whose appearance plausibly supported either sweet or savoury expectations, Yeomans et al. (2008) demonstrated that violating a strong sweet expectation leads to sharp reductions in pleasantness alongside exaggerated perception of unexpected sensory attributes, particularly saltiness. Subsequent work reinforces the prominence of contrast effects under strong expectancy violations, especially when verbal and visual cues are incongruent, demonstrating that expectancy disconfirmation can simultaneously reduce liking and amplify unexpected sensory qualities (Piqueras-Fiszman and Spence, 2015). In sum, this literature suggests that expectancy violation can affect both hedonic evaluation and the weighting of specific sensory attributes, but it remains unclear how general these effects are across flavour trajectories and whether sensory and hedonic contrast necessarily unfold symmetrically.

### 1.4 The present study

This study builds directly on the smoked salmon ice-cream paradigm while addressing three critical limitations that constrain the generality and mechanistic interpretation of expectancy-driven contrast effects. First, whereas Yeomans et al. (2008) combined visual appearance with verbal labelling to induce expectations, the current study isolates the contribution of linguistic labels alone. Pre-taste evaluations were elicited from written descriptions in the absence of visual cues, allowing a direct test of whether labels themselves are sufficient to generate anticipatory aversion. Demonstrating a label-only driven shift in pleasantness would confirm that expectancy violations do not require visual incongruence but can arise purely from semantic framing.

Second, the study introduces a bidirectional design that directly measures expectancy violations in both directions. Prior paradigms primarily examined violations in one polarity (*e.g.*, sweet expectation followed by savoury taste). Here, we reverse the semantic and hedonic frames of reference. Some foods were labelled as sweet products, strongly associated with pleasantness and sugar, yet delivered a dominant savoury or umami profile, including animal or fermented notes that are particularly jarring when unexpected. Conversely, other foods were labelled as savoury, associated with saltiness, meatiness and umami, yet delivered a dominant sweet taste. This design directly quantifies the mismatch between expected and actual flavour profiles in both directions (sweet-to-salty; salty-to-sweet), ensuring that discrepancies are sufficiently large to elicit contrast rather than assimilation. By measuring both sensory attributes (sweetness, saltiness) and hedonic responses before and after tasting, the study provides a direct test of the expectancy-contrast mechanism across inverted semantic frames.

Third, the study substantially increases stimulus breadth to test generality. Rather than relying on a single highly novel product, we examine ten foods spanning opposing flavour trajectories and include a neutral labelling condition. This broader stimulus set allows us to determine whether aversion arises specifically from expectancy violation rather than from a simple sweet-savoury bias. The inclusion of multiple items and a neutral labelling condition ensures that any observed contrast effects reflect a robust expectancy-driven mechanism rather than an idiosyncratic property of a single food product. Together, the benchmark replication and the ten-item bidirectional design provide a stronger test of whether misleading labels alone can generate systematic sensory and hedonic contrast effects across flavour directions and food types.

The primary hypothesis was that misleading labels would generate strong anticipatory expectancy effects and subsequent contrast-based re-evaluation in both flavour trajectories. Specifically, we expected Condition × Time interactions for pleasantness and for the key sensory dimensions of sweetness and saltiness, reflecting a mismatch between label-based expectations and tasted experience. We further expected that these effects would be strongest in the Unexpected condition, with Neutral labels producing intermediate responses. There was no strong directional prediction that hedonic contrast would necessarily be greater in the Sweet-to-Salty trajectory than in the Salty-to-Sweet trajectory. Similarly, although we expected expectancy-sensitive sensory reweighting in both directions, whether sweetness and saltiness would show fully symmetric adjustment across trajectories was left open. The present design thus tests both a confirmatory prediction, namely, that misleading labels induce contrast-based re-evaluation, and a broader comparative question concerning the directional symmetry of hedonic and sensory updating.

## 2 Methodology

### 2.1 Participants

Participants were 40 Mandarin-dominant adults recruited from the University of Auckland community. All participants reported normal taste function and confirmed that they had no cold, flu or allergies at the time of testing that could affect taste perception. Volunteers with known food allergies, diabetes or eating disorders were excluded. The study was described as unsuitable for vegetarians due to the inclusion of animal-based ingredients in some stimuli. Participants ranged in age from 20 to 36 years (*M =* 27.90, *SD =* 3.54). They were naïve to the specific aims of the study and were informed only that the experiment involved the evaluation of food products that were safe to consume but might taste unusual. All participants provided informed consent prior to participation and were compensated for their time.

The study protocol was approved by the University of Auckland Ethics Committee (#25922) and was conducted in accordance with institutional ethical guidelines. The same 40 participants also completed the smoked salmon ice-cream benchmark under the same testing conditions.

### 2.2 Test food products

**Fig. 1:**
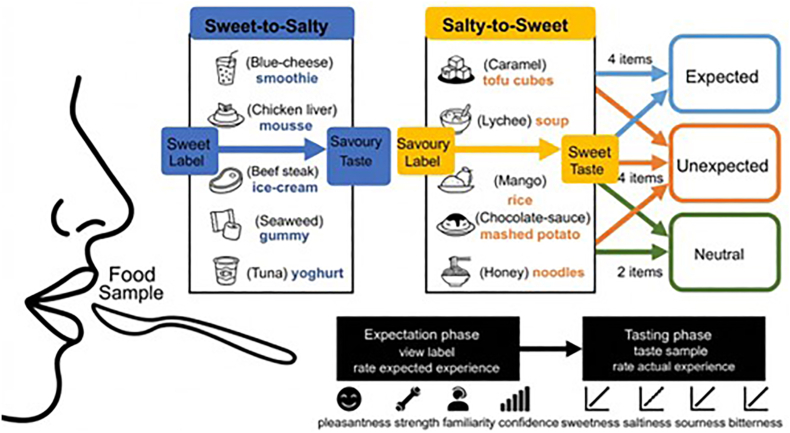
Schematic overview of the experimental design and procedure.

The main experiment comprised ten target foods. Five items followed a Sweet-to-Salty trajectory (Blue-cheese smoothie, Chicken liver mousse, Beef steak ice-cream, Seaweed gummy, Tuna yoghurt), in which products labelled as sweet delivered dominant savoury or umami profiles. Five followed a Salty-to-Sweet trajectory (Caramel tofu cubes, Lychee soup, Mango rice, Chocolate-sauce mashed potato, Honey noodles), in which products labelled as savoury delivered dominant sweet tastes (Fig. 1). In addition, the same participants completed a smoked salmon ice-cream benchmark trial, prepared according to Yeomans et al. (2008), to verify that the present label-only procedure reproduced the classic expectancy-contrast pattern under our testing conditions. The benchmark stimulus was analysed separately from the ten-item dataset and is reported first as a procedural validation.

All food ingredients used to produce the model foods were commercial foodstuffs purchased from supermarkets or food ingredient suppliers. All model foods were made within 24 h of testing apart from the smoked salmon ice cream and the beef steak ice cream, which were prepared in advance and stored frozen until use. The *smoked salmon ice-cream* benchmark followed Yeomans et al. (2008), using 2 L full-fat UHT milk, 708 ml UHT cream, 832 g maltodextrin, 28 g table salt, and 10.6 g MSG to make the bulk savoury ice cream. Then we took 1 kg savoury ice cream and added 228 g smoked salmon, following the proportion of 18.5% smoked salmon in the smoked salmon ice cream used in the original recipe (Yeomans et al., 2008). *Tuna yoghurt* was prepared from 50 g of canned tuna in spring water (drained) and 220 g of full-fat Greek-style unsweetened yoghurt blended on high speed for 2 min (Vitamix Explorian Blender E320). *Blue cheese smoothie* was prepared from 50 g Danish creamy blue cheese, 165 mL full-fat UHT milk and 55 mL UHT cream blended on low speed for 2 min. For *Beef steak ice cream*, the Smoked salmon ice cream method (above) was used with the following ingredients and quantities; 1 L full-fat UHT milk, 354 mL UHT cream, 416 g maltodextrin (Equagold maltodextrin powder DE 10-12), 407.5 g canned corned beef, 14 g table salt and 5.3 g MSG. The corned beef was blended prior to mixing through the ice cream on high speed for 1 min to create a homogenous paste. The ice cream was stored in an airtight container at −18 °C until use (within 2 weeks). *Seaweed gummy* was prepared from 1 standard sized sheet of toasted nori (seaweed), crushed in a mortar and pestle and then soaked in 100 mL room temperature water for 2 h and then sieved to remove the seaweed particles. 10 g of granulated gelatine was dispersed onto the surface of 30 mL the seaweed-water to bloom. 220 g of warm water (40 °C) was added to this mixture with 50 mL of green food colouring and mixed until the gelatine dissolved completely. The mixture was poured into silicone chocolate moulds and kept at 4 °C for 12 h. For *Chicken liver mousse, c*ommercial ‘farmhouse style’ chicken liver mousse was cut into 2 cm × 1.2 cm cubes prior to serving. For *Caramel tofu cubes*, firm tofu was cut into 1.5 cm × 1.5 cm cubes. The tofu was lightly pan-fried in 15 g of canola oil and coated with 30 g of commercial caramel sauce. For *Lychee soup*, canned lychees (in natural juice) were pureed; 100 mL of the puree was combined with 100 mL water and served warm. For *Honey noodles*, 5 g dry ramen noodles were cooked, drained, and coated with 5 g of honey. For *Mango rice*, 200 g of medium grain white rice and 300 g of water was combined and heated in a rice cooker for 20 min in water and topped, for each serving, with 6 g of mango puree prepared from canned mango (in natural juice). For serving, 30 g of cooked rice was topped with 6 g of mango puree. For *Chocolate-sauce mashed potato*, 40 g of potato flakes were rehydrated in 250 mL of boiling water and 1.25 g table salt. The chocolate sauce was made by combining 3:1 commercial chocolate sauce and caramel sauce. For each serving, 12 g of mashed potato were topped with 4 g of chocolate sauce. Samples were cooled to and served at room temperature.

The target foods were selected primarily to operationalise the experimental logic of the study rather than to represent any one culinary tradition. Specifically, the items were chosen to instantiate two clearly opposed flavour trajectories, Sweet-to-Salty and Salty-to-Sweet, and to produce strong mismatches between the flavour profile implied by the label and the dominant flavour profile experienced during tasting. We also aimed to increase stimulus breadth relative to the original smoked salmon ice-cream paradigm by including multiple food types and formats, so that any observed effects could not be attributed to a single unusual product. The selection was not intended as a direct test of foods specific to Mandarin-speaking participants' cultural background, although all labels and instructions were presented in Mandarin and the items were chosen to be sufficiently interpretable as named food products for this participant group. At the same time, we acknowledge that participants’ cultural background may have influenced baseline familiarity, plausibility and acceptability of some items, and thus may have contributed to item-level variation in the observed responses.

### 2.3 Procedure

Participants attended a single tasting session conducted individually in a quiet, green-lit sensory testing booth. During this session, they completed the ten-item bidirectional task and, in a separate benchmark block, evaluated the smoked salmon ice-cream stimulus. The benchmark block used the same label-only expectation procedure, the same rating scales and the same sensory testing environment as the main experiment but was analysed separately because its purpose was procedural validation rather than inclusion in the multi-item trajectory analyses. All instructions and written materials were presented in Mandarin, participants’ native language. Participants were instructed to refrain from eating or smoking and to consume only water for 1 h prior to testing, and confirmed upon arrival that they had no cold, flu or allergies that could affect taste perception. Prior to participation, volunteers confirmed that they had no known food allergies, diabetes or eating disorders. They were informed that the food items used in the experiment might contain traces of gluten, milk, nuts, offal/liver, fish and related ingredients. Individuals reporting extreme dislike or strong aversion to any of these categories were excluded to minimise confounds unrelated to expectancy violation. At the start of the session, participants completed baseline ratings of current hunger (“How hungry are you now?” 1 = not at all hungry, 9 = extremely hungry) and affective state. Following Yeomans et al. (2008), participants indicated the extent to which they currently felt clear-headed, drowsy, thirsty, lively, calm, full, nervous, relaxed, hungry and nauseous, using 9-point scales. During tasting, participants were instructed to swallow the sample before responding and to provide their ratings immediately after swallowing. They were encouraged to use the full range of the 9-point scales and were explicitly told “Please use the whole scale from 1 to 9. Do not hesitate to use the endpoints if they reflect your experience”.

The critical manipulation involved the verbal label used to describe each food item. Each participant evaluated all ten foods within a single session. For each participant, label conditions were assigned such that four items appeared in the Expected (congruent) condition, four in the Unexpected (incongruent) condition, and two in a Neutral condition. This 4:4:2 allocation was chosen to maximise sensitivity for the theoretically central contrast between congruent and incongruent labelling while retaining a neutral reference condition. As a consequence, the Neutral condition is represented by fewer observations than the Expected and Unexpected conditions, and its estimates should therefore be interpreted somewhat more cautiously. In the Expected condition, the label matched the actual dominant flavour direction of the product (*e.g.*, a sweet-labelled sweet-tasting food or a savoury-labelled savoury-tasting food). In the Unexpected condition, the label implied the opposite flavour direction (*e.g.*, a sweet label for a savoury-dominant product, or a savoury label for a sweet-dominant product), creating a strong expectancy violation. In the Neutral condition, the item was presented with an uninformative label (*e.g. Food item 66*), providing no indication of flavour direction. The Neutral condition was intended to serve as a low-information baseline rather than a fully expectation-free condition. Although the generic label did not signal a specific flavour direction, participants may nevertheless have formed weak default expectations based on the fact that the stimulus was described as a food item. Label assignment was counterbalanced using four Latin-square schedules so that each of the ten items appeared equally often in the Expected, Unexpected, and Neutral conditions across participants. This design ensured that any observed effects could be attributed to expectancy manipulation rather than intrinsic properties of specific stimuli.

Each trial consisted of two phases. In the Expectation phase, participants viewed the written label and rated their expected experience of the product based solely on the label information. In the Tasting phase, participants were served a single teaspoon-sized portion (approximately 10 g) of the food item, along with water for palate cleansing, and repeated the same ratings based on their actual sensory experience. Participants were instructed to place the entire sample in their mouth, swallow and provide their ratings immediately after swallowing. Re-tasting was not permitted. Ratings were made on 9-point scales and included pleasantness, perceived strength, familiarity, confidence and flavour attributes (sweetness, saltiness, sourness, bitterness).

In the label-exposure phase, participants were presented with written food labels only (without visual cues) and asked to provide anticipatory ratings. For each food item, labels appeared in one of three expectancy conditions: Expected, Unexpected, or Neutral. In the Sweet-to-Salty trajectory, the Expected labels were Tuna yoghurt (吞拿鱼酸奶, tūn ná yú suān nǎi), Blue-cheese smoothie (蓝纹芝士奶昔, lán wén zhī shì nǎi xī), Chicken liver mousse (鸡肝慕斯, jī gān mù sī), Beef steak ice-cream (牛排冰激凌, niú pái bīng jī líng), and Seaweed gummy (海苔软糖, hǎi tái ruǎn táng). Their corresponding Unexpected labels were respectively: Strawberry yoghurt (草莓酸奶, cǎo méi suān nǎi), Blueberry smoothie (蓝莓奶昔, lán méi nǎi xī), Chocolate mousse (巧克力慕斯, qiǎo kè lì mù sī), Cookie dough ice-cream (饼干碎冰激凌, bǐng gān suì bīng jī líng), and Apple gummy (苹果软糖, píng guǒ ruǎn táng). In the Salty-to-Sweet trajectory, the Expected labels were Chocolate-sauce mashed potato (巧克力酱土豆泥, qiǎo kè lì jiàng tǔ dòu ní), Mango rice (芒果饭, máng guǒ fàn), Honey noodles (蜂蜜拌面, fēng mì bàn miàn), Caramel tofu cubes (焦糖豆腐块, jiāo táng dòu fu kuài), and Lychee soup (荔枝汤, lì zhī tāng). Their Unexpected counterparts were respectively Gravy mashed potato (肉汁土豆泥, ròu zhī tǔ dòu ní), Curry rice (咖喱饭, gā lǐ fàn), Soy sauce noodles (酱油拌面, jiàng yóu bàn miàn), Fried tofu cubes (煎豆腐块, jiān dòu fu kuài), and Pork rib soup (排骨汤, pái gǔ tāng). In the Neutral condition, all items were labelled generically as Food xxx (食物 xxx, shí wù xxx), where “xxx” was replaced by a number to avoid conveying any flavour-specific expectations.

### 2.4 Analyses

Analyses were conducted separately for the smoked salmon ice-cream benchmark and for the ten-item bidirectional dataset. The smoked salmon ice-cream data were analysed as a procedural replication of the classic expectancy-contrast effect. For this dataset, linear mixed-effects models tested the effects of Label, Time (before tasting vs. after tasting), and their interaction on pleasantness, sweetness and saltiness ratings. The ten target foods were analysed in two trajectory-specific datasets, Sweet-to-Salty and Salty-to-Sweet, to allow directional comparisons without collapsing across qualitatively different flavour transitions. Within each trajectory, separate linear mixed-effects models were fitted for each dependent variable. Fixed effects included Condition (Expected, Neutral, Unexpected), Time (before tasting vs. after tasting), and their interaction, together with perceived strength, familiarity, confidence, hunger, and mood as covariates. Treatment coding was used for categorical predictors, with Expected and After-taste serving as the reference levels. Random intercepts were specified for Participant and Item to account for repeated observations within individuals and stimuli. Where more complex random-effects structures led to singular fits, the random structure was simplified to retain only intercepts. Models were fitted by maximum likelihood using the *lme4* package in R (Bates et al., 2015), with the *bobyqa* optimizer and increased iteration limits where needed. Fixed effects were evaluated using Satterthwaite-approximated degrees of freedom as implemented in *lmerTest* (Kuznetsova et al., 2017). Follow-up comparisons were conducted using estimated marginal means from the *emmeans* package (Lenth et al., 2026), with Holm correction applied to pairwise contrasts. In interpreting the models, the Time coefficient represents the before-after difference in the reference condition, while the Condition × Time interaction terms indicate how this shift differs in the Neutral and Unexpected conditions. Because the Neutral condition contained fewer trials per participant than the Expected and Unexpected conditions, it was treated primarily as a reference baseline rather than as a condition matched in power to the two critical expectancy conditions. Model convergence and residual patterns were inspected in all cases. Also, because the smoked salmon stimulus served as a benchmark rather than as part of the ten-item extension, it was not pooled with the main item set in any omnibus model. For consistency across analyses, fixed-effect results are reported in the format *β*, *SE*, *t*, and *p*, random effects are described as random intercepts for Participant and Item, and inferential statistics are based on Satterthwaite-approximated degrees of freedom.

## 3 Results

### 3.1 Benchmark replication of the smoked salmon ice-cream effect

Before turning to the ten-item bidirectional dataset, we analysed the smoked salmon ice-cream benchmark separately as a procedural validation. This benchmark was not included in the ten-item trajectory analyses. Its purpose was to test whether the present label-only procedure reproduced the established expectancy-contrast pattern reported by Yeomans et al. (2008). The classic expectancy-contrast pattern was replicated. For pleasantness, ratings were substantially higher before tasting under the *ice-cream* label than after tasting, whereas the decline was smaller under the *savoury mousse* label. A mixed-effects model revealed a robust main effect of Time (*β =* 2.57, *SE =* 0.65, *t =* 3.95, *p <* .001) qualified by a significant Label × Time interaction (*β =* −1.91, *SE =* 0.90, *t =* −2.12, *p* = .036), indicating a larger pre-post drop in pleasantness when the product was labelled as *ice-cream*. Table 1 provides the descriptive means and standard deviations for the smoked salmon benchmark across label conditions and time points, allowing direct inspection of the size and direction of the replicated expectancy-contrast effect alongside the mixed-effects model results.

**Table 1:** Descriptive summary of the smoked salmon ice-cream benchmark by label and time point.

Dependent variable	Label condition	Before tasting M (SD)	After tasting M (SD)	Δ (Before − After)
Pleasantness	Ice-cream	7.42 (1.66)	3.58 (2.62)	3.84
Pleasantness	Savoury mousse	4.65 (1.93)	3.65 (2.30)	1.00
Sweetness	Ice-cream	7.05 (1.97)	1.65 (1.17)	5.40
Sweetness	Savoury mousse	3.82 (2.50)	1.88 (1.45)	1.94
Saltiness	Ice-cream	1.90 (1.30)	6.20 (2.21)	−4.30
Saltiness	Savoury mousse	5.00 (2.31)	6.75 (1.88)	−1.75

A parallel pattern emerged for sweetness and saltiness. Sweetness ratings showed a strong Time effect (*β =* 4.78, *SE =* 0.56, *t =* 8.54, *p <* .001) with a significant Label × Time interaction (*β =* −3.05, *SE =* 0.77, *t =* −3.96, *p <* .001), reflecting a sharper reduction in perceived sweetness under the ice-cream label. Saltiness showed the inverse pattern, with ratings increasing after tasting (*β =* −3.87, *SE =* 0.58, *t =* −6.67, *p <* .001) and a significant Label × Time interaction (*β =* 2.00, *SE =* 0.81, *t =* 2.47, *p *= .015), indicating a stronger post-taste increase in saltiness under the incongruent label. Together, these results reproduce the established expectancy-contrast effect. Anticipatory sweetness and pleasantness under a sweet label were sharply reduced following tasting, accompanied by enhanced perception of saltiness. Having confirmed that the present procedure reproduces the benchmark expectancy-contrast effect, we next report the analyses of the independent ten-item bidirectional dataset.

### 3.2 Sweet-to-Salty trajectory

#### 3.2.1 Hedonic contrast (pleasantness)

**Fig. 2:**
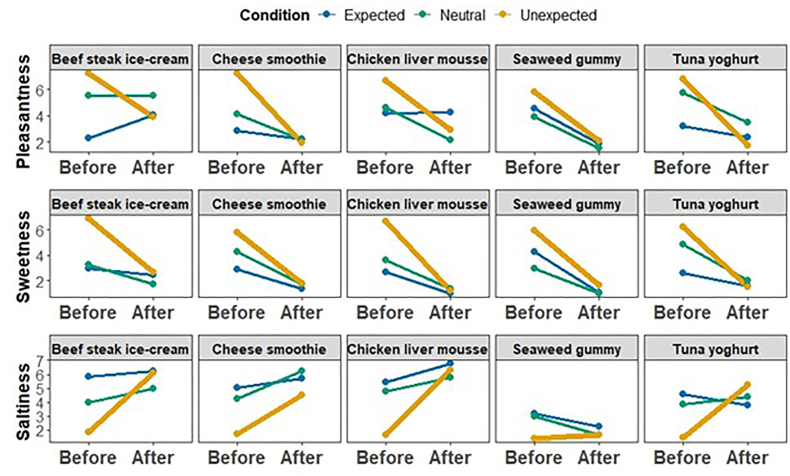
Expectancy violation in the Sweet-to-Salty trajectory, showing hedonic contrast and sensory reweighting across five items. Mean ratings (aggregated across participants) are shown separately for five initially sweet-labelled items that were revealed to be savoury/salty upon tasting. The top row displays pleasantness ratings, the middle row sweetness ratings and the bottom row saltiness ratings. Within each panel, lines connect pre-ingestive expectations (Before) with post-ingestive evaluations (After) for Expected, Neutral and Unexpected label conditions.

Descriptively, as shown in Fig. 2 and Table 2, pleasantness ratings decreased from before tasting to after tasting across all expectancy conditions. In the Expected condition, pleasantness declined from *M =* 3.43 (*SD =* 2.52) before tasting to *M =* 2.95 (*SD =* 2.20) after tasting (Δ = 0.48). In the Neutral condition, ratings dropped from *M =* 4.78 (*SD =* 2.20) before tasting to *M =* 2.95 (*SD =* 2.42) after tasting (Δ = 1.83). The most pronounced decrease was observed in the Unexpected condition, where pleasantness fell sharply from *M =* 6.71 (*SD =* 2.23) before tasting to *M =* 2.51 (*SD =* 2.10) after tasting (Δ = 4.20). This graded pattern indicates that the magnitude of the decline in pleasantness increased as expectancy became less aligned, with the largest re-evaluation occurring under Unexpected labeling. A linear mixed-effects model predicting pleasantness (Condition × Time, with Strong, Familiar, Confidence, Hunger and mood as covariates, and random intercepts for Participant and Item) revealed a significant main effect of Time, with before-taste ratings reliably higher than after-taste ratings (*β =* 0.62, *SE =* 0.30, *t =* 2.08, *p* = .038). Crucially, this effect was qualified by a significant Condition × Time interaction. Relative to the Expected condition, the before-after shift was significantly larger in the Neutral condition (*β =* 1.16, *SE =* 0.52, *t =* 2.23, p = .027) and in the Unexpected condition (*β =* 1.99, *SE =* 0.45, *t =* 4.45, *p <* .001), indicating that the magnitude of the re-evaluation differed across expectancy contexts. Planned contrasts using estimated marginal means (Holm-adjusted) confirmed that the decline from before to after tasting was significant in all three conditions (Expected: Δ = 0.62, *SE =* 0.30, *t =* 2.04, *p* = .043; Neutral: Δ = 1.78, *SE =* 0.46, *t =* 3.85, *p <* .001; Unexpected: Δ = 2.61, *SE =* 0.35, *t =* 7.57, *p <* .001), with the largest decline observed in the Unexpected condition. To clarify where expectancy effects emerged, condition contrasts were examined separately at each time point. At the after-taste stage, pleasantness ratings did not differ significantly between conditions (all *p*s = 1.00 after Holm correction), indicating convergence following sensory input. In contrast, at the before-taste stage, significant differences were observed. Pleasantness in the Expected condition was lower than in the Neutral condition (Δ = −1.11, *SE =* 0.39, *t =* −2.85, *p* = .009) and the Unexpected condition (Δ = −1.82, *SE =* 0.34, *t =* −5.44, *p <* .001). The difference between Neutral and Unexpected did not reach significance after correction (*p =* .085). These findings indicate that expectancy primarily shaped anticipatory pleasantness, with sensory experience attenuating these contextual differences post-ingestion. Inclusion of covariates (*e.g.*, familiarity, perceived strength, hunger, mood) did not alter the pattern of significant interactions. Overall, the results show that expectancy context modulates anticipatory pleasantness and shapes the magnitude of tasting-related re-evaluation, with sensory experience attenuating these contextual differences post-ingestion.

**Table 2:** Estimated marginal means (EMMs), before-after differences, and Holm-adjusted contrasts (Sweet-to-Salty). Δ values are computed as Before - After. Positive values indicate decreases after tasting, negative values indicate increases, p-values are Holm-adjusted, ns = not significant.

DV	Condition	Before M (SD)	After M (SD)	Δ (Before − After)	p (Holm, Δ)
**Pleasantness**	Expected	3.43 (2.52)	2.95 (2.20)	0.62	0.043
	Neutral	4.78 (2.20)	2.95 (2.42)	1.78	<0.001
	Unexpected	6.71 (2.23)	2.51 (2.10)	2.61	<0.001
**Sweetness**	Expected	3.10 (2.34)	1.50 (1.21)	1.57	<0.001
	Neutral	3.80 (1.95)	1.58 (0.96)	2.22	<0.001
	Unexpected	6.31 (2.16)	1.79 (1.52)	4.52	<0.001
**Saltiness**	Expected	4.81 (2.29)	4.95 (2.62)	−0.18	ns
	Neutral	3.98 (1.98)	4.60 (2.49)	−0.62	ns
	Unexpected	1.62 (1.40)	4.78 (2.70)	−3.16	<0.001

DV	Time	Significant contrasts
Pleasantness	Before	Expected < Neutral (p = .009); Expected < Unexpected (*p <* .001); Neutral ≈ Unexpected
	After	All ns
Sweetness	Before	Unexpected > Expected (*p <* .001); Unexpected > Neutral (*p <* .001); Expected ≈ Neutral
	After	All ns
Saltiness	Before	Unexpected < Expected (*p <* .001); Unexpected < Neutral (*p <* .001); Expected ≈ Neutral
	After	All ns

#### 3.2.2 Sensory reweighting: sweetness

Sweetness ratings (Fig. 2) showed a strong anticipatory effect across expectancy conditions. In the Expected condition, sweetness decreased from *M =* 3.10 (*SD =* 2.34) before tasting to *M =* 1.50 (*SD =* 1.21) after tasting (Δ = 1.60). In the Neutral condition, ratings declined from *M =* 3.80 (*SD =* 1.95) before tasting to *M =* 1.58 (*SD =* 0.96) after tasting (Δ = 2.22). The most pronounced decrease was observed in the Unexpected condition, where sweetness dropped sharply from *M =* 6.31 (*SD =* 2.16) before tasting to *M =* 1.79 (*SD =* 1.52) after tasting (Δ = 4.52). This descriptive pattern indicates a strong expectancy-sensitive anticipatory component, with the largest re-evaluation under Unexpected labeling. Consistent with this pattern, the mixed-effects model revealed a robust main effect of Time (Before-After), with sweetness rated significantly higher before tasting than after tasting (*β =* 1.57, *SE =* 0.24, *t =* 6.44, *p <* .001). Crucially, this effect was qualified by a significant Condition × Time interaction for the Unexpected condition. Relative to the Expected condition, the before-after difference was significantly larger in the Unexpected condition (*β =* 2.29, *SE =* 0.37, *t =* 6.23, *p <* .001). In contrast, the interaction for the Neutral condition was not significant (*β =* 0.37, *SE =SE =* 0.43, *t =* 0.87, *p =* .385), indicating that the magnitude of re-evaluation in the Neutral condition did not reliably differ from the Expected condition once covariates were accounted for. Importantly, there were no significant main effects of Condition independent of Time (Neutral: *p =* .83; Unexpected: *p =* .11), indicating that sweetness ratings did not differ reliably across expectancy contexts at the after-taste stage. Among the covariates, perceived strength was negatively associated with sweetness (*β =* −0.14, *SE =* 0.046, *t =* −3.04*, p =* .003), whereas familiarity showed a positive association (*β =* 0.16, *SE =* 0.038, *t =* 4.12, *p <* .001). Confidence showed a marginal negative association (*β =* −0.12, *SE =* 0.06, p = .050), while hunger did not significantly predict sweetness ratings (*p* = .51). Mood categories did not alter the central Condition × Time pattern. Overall, sweetness judgments exhibited a pronounced expectancy-sensitive anticipatory effect, with sensory experience substantially attenuating condition-based differences after tasting, particularly under Unexpected labeling.

#### 3.2.3 Sensory reweighting: saltiness

Saltiness ratings (Fig. 2) showed the inverse pattern to sweetness, with the strongest expectancy modulation emerging in the Unexpected condition. Descriptively, saltiness ratings were relatively stable in the Expected condition, increasing slightly from *M =* 4.81 (*SD =* 2.29) before tasting to *M =* 4.95 (*SD =* 2.62) after tasting (After − Before Δ = +0.14). In the Neutral condition, saltiness increased modestly from *M =* 3.98 (*SD =* 1.98) before tasting to *M =* 4.60 (*SD =* 2.49) after tasting (Δ = +0.62). In contrast, the Unexpected condition showed a pronounced shift. Anticipatory saltiness ratings were very low (*M =* 1.62, *SD =* 1.40) but increased sharply after tasting (*M =* 4.78, *SD =* 2.70), yielding a substantial re-evaluation (Δ = +3.16). Thus, expectancy violation produced a dramatic underestimation of saltiness prior to tasting. The mixed-effects model corroborated this pattern. In the reference (Expected) condition, there was no significant main effect of Time (*β =* −0.18, *SE =* 0.27, *t =* −0.66, *p* = .51), indicating no reliable change from before to after tasting under accurate labeling. Crucially, this effect was qualified by a significant Condition × Time interaction for the Unexpected condition. Relative to the Expected condition, the before–after shift was significantly larger in the Unexpected condition (*β =* −2.43, *SE =* 0.41, *t =* −5.89, *p <* .001), reflecting the substantial expectancy-violation effect. In contrast, the Neutral × Time interaction was not significant (*β =* −0.57, *SE =* 0.48, *t =* −1.20, *p* = .23), indicating that the modest shift in the Neutral condition did not reliably differ from the Expected condition once covariates were included. Among covariates, perceived strength was a strong positive predictor of saltiness (*β =* 0.49, *SE =* 0.05, *t =* 9.24, *p <* .001). Confidence showed a small but significant negative association (*β =* −0.15, *SE =* 0.07, *t =* −2.13, p = .034), whereas familiarity was not a significant predictor (*p* = .45). Hunger did not significantly predict saltiness ratings (*p* = .23). Several mood categories showed individual effects, but these did not alter the central Condition × Time pattern. Overall, saltiness judgments were largely stable under Expected and Neutral labeling but exhibited a strong expectancy-violation effect under Unexpected labelling. Anticipatory saltiness was dramatically underestimated and then sharply corrected following sensory experience. This pattern mirrors the sweetness results, confirming that expectancy primarily shaped anticipatory sensory judgments, with bottom-up gustatory input restoring accuracy post-ingestion.

### 3.3 Salty-to-Sweet trajectory

#### 3.3.1 Hedonic contrast (pleasantness)

**Fig. 3:**
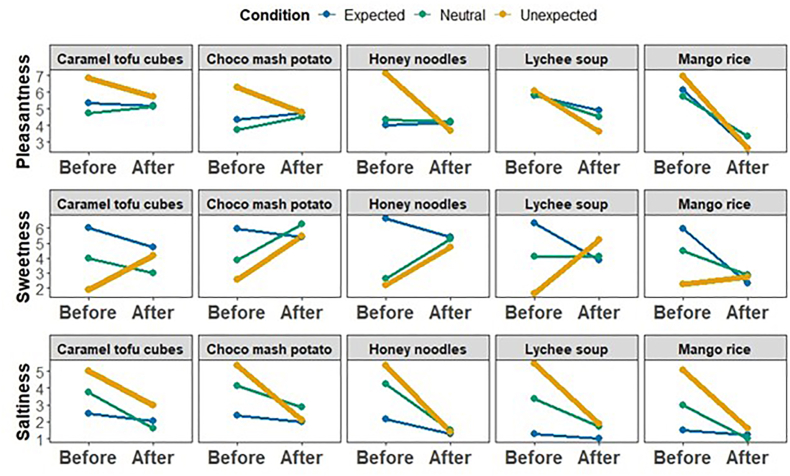
Expectancy violation in the Salty-to-Sweet trajectory, showing hedonic contrast and sensory reweighting across five items. Mean ratings (± aggregation across participants) are shown separately for five initially salty-labelled items that were revealed to be sweet upon tasting. The top row displays pleasantness ratings, the middle row sweetness ratings and the bottom row saltiness ratings. Within each panel, lines connect pre-ingestive expectations (Before) with post-ingestive evaluations (After) for Expected, Neutral and Unexpected label conditions.

Descriptively, as shown in Fig. 3 and Table 3, pleasantness ratings decreased from before tasting to after tasting across all expectancy conditions, although the magnitude of this decline was more moderate than in the Sweet-to-Salty direction. In the Expected condition, pleasantness declined from *M =* 5.14 (*SD =* 2.28) before tasting to *M =* 4.34 (*SD =* 2.13) after tasting (Δ = 0.80). In the Neutral condition, ratings decreased from *M =* 4.90 (*SD =* 1.98) before tasting to *M =* 4.35 (*SD =* 1.97) after tasting (Δ = 0.55). In the Unexpected condition, pleasantness fell from *M =* 6.66 (*SD =* 1.91) before tasting to *M =* 4.10 (*SD =* 2.35) after tasting (Δ = 2.56). Thus, while anticipatory pleasantness was again highest under Unexpected labeling, tasting eliminated condition differences in both directions. However, unlike the Sweet-to-Salty dataset, the present direction did not show a significant Condition × Time interaction, indicating that expectancy modulated overall anticipation but did not differentially amplify the magnitude of re-evaluation. The mixed-effects model revealed a significant main effect of Time, with before-taste ratings reliably higher than after-taste ratings (*β =* 0.78, *SE =* 0.28, *t =* 2.75, *p =* .006). However, unlike in the Sweet-to-Salty dataset, the Condition × Time interaction was not significant (Neutral: *β =* 0.21, *SE =* 0.47, *t =* 0.44, *p* = .661; Unexpected: *β =* 0.54, *SE =* 0.40, *t =* 1.36, *p* = .174), indicating that the magnitude of the before-after shift did not differ reliably across expectancy contexts. Follow-up contrasts confirmed that the before-after decline was significant in all three conditions: Expected (Δ = 0.78, *SE =* 0.30, *p* = .009), Neutral (Δ = 0.98, *SE =* 0.43, p = .023), and Unexpected (Δ = 1.32, *SE =* 0.32, *p <* .001). Importantly, no significant differences between conditions were observed either at the before-taste or after-taste stage (all Holm-adjusted *p*s ≥ 0.508), indicating substantial convergence across expectancy contexts. Among covariates, perceived strength was positively associated with pleasantness (*β =* 0.15, *SE =* 0.052, *t =* 2.87, *p* = .004), and familiarity showed a strong positive association (*β =* 0.44, *SE =* 0.041, *t =* 10.63, *p <* .001). Confidence and hunger did not significantly predict pleasantness. Overall, the Salty-to-Sweet direction shows a general pre–post decline in pleasantness, but without the graded expectancy-sensitive interaction observed in the Sweet-to-Salty dataset. This pattern is suggestive of a possible trajectory difference, but because the two directions were analysed in separate models, it should not be taken as formal evidence that expectancy-driven re-evaluation differs significantly across flavour trajectories.

**Table 3:** Estimated marginal means (EMMs), before-after differences, and Holm-adjusted contrasts (Salty-to-Sweet). Δ values are computed as Before - After. Positive values indicate decreases after tasting, negative values indicate increases, p-values are Holm-adjusted, ns = not significant.

DV	Condition	Before M (SD)	After M (SD)	Δ (Before − After)	p (Holm, Δ)
Pleasantness	Expected	5.14 (2.28)	4.34 (2.13)	0.78	0.009
	Neutral	4.90 (1.98)	4.35 (1.97)	0.98	0.023
	Unexpected	6.66 (1.91)	4.10 (2.35)	1.32	<0.001
Sweetness	Expected	6.16 (2.22)	4.34 (2.41)	1.60	<0.001
	Neutral	3.82 (1.57)	4.30 (2.22)	−0.67	0.132
	Unexpected	2.10 (1.67)	4.46 (2.44)	−2.80	<0.001
Saltiness	Expected	1.98 (1.35)	1.52 (1.15)	0.22	0.341
	Neutral	3.70 (2.21)	1.75 (1.37)	1.68	<0.001
	Unexpected	5.22 (1.78)	2.00 (1.58)	2.82	<0.001

DV	Time	Significant contrasts
Pleasantness	Before	All ns
	After	All ns
Sweetness	Before	Expected > Neutral (*p <* .001); Expected > Unexpected (*p <* .001); Neutral > Unexpected (*p <* .001)
	After	All ns
Saltiness	Before	Expected < Neutral (*p <* .001); Expected < Unexpected (*p <* .001); Neutral < Unexpected (*p <* .001)
	After	Expected < Unexpected (p = .047); other contrasts ns

#### 3.3.2 Sensory reweighting: sweetness

Sweetness ratings (Fig. 3) showed a markedly different pattern compared to the Sweet-to-Salty direction, with expectancy effects emerging primarily at the anticipatory stage and in opposite directions across conditions. In the Expected condition, sweetness decreased from *M =* 6.16 (*SD =* 2.22) before tasting to *M =* 4.34 (*SD =* 2.41) after tasting (Δ = 1.82). In contrast, the Neutral condition showed a slight increase in sweetness from *M =* 3.82 (*SD =* 1.57) before tasting to *M =* 4.30 (*SD =* 2.22) after tasting (Δ = −0.48). The most pronounced reversal was observed in the Unexpected condition, where sweetness increased sharply from *M =* 2.10 (*SD =* 1.67) before tasting to *M =* 4.46 (*SD =* 2.44) after tasting (Δ = −2.36). Thus, while sweetness declined in the Expected condition following tasting, it increased substantially under Unexpected labeling, indicating strong expectancy-driven contrast effects. The mixed-effects model revealed a significant main effect of Time in the reference condition (*β =* 1.60, *SE =* 0.30, *t =* 5.40, *p <* .001), confirming higher sweetness ratings before than after tasting in the Expected condition. Crucially, this effect was strongly qualified by significant Condition × Time interactions. Relative to Expected, the before-after shift was significantly reversed in the Neutral condition (*β =* −2.27, *SE =* 0.50, *t =* −4.58, *p <* .001) and even more dramatically in the Unexpected condition (*β =* −4.40, *SE =* 0.41, *t =* −10.62, *p <* .001). Follow-up contrasts clarified this pattern. In the Expected condition, sweetness was significantly higher before than after tasting (Δ = 1.60, *SE =* 0.30, *p <* .001). In contrast, the Neutral condition did not show a statistically reliable before-after difference (Δ = −0.67, *SE =* 0.44, p = .132). In the Unexpected condition, sweetness was significantly higher after than before tasting (Δ = −2.80, *SE =* 0.33, *p <* .001). Importantly, no condition differences were observed at the after-taste stage (all Holm-adjusted ps = 1.00), indicating convergence once tasting occurred. However, at the before-taste stage, all three conditions differed significantly from one another (all *p*s < .001), with Expected showing the highest anticipatory sweetness and Unexpected the lowest. Among the covariates, perceived strength was positively associated with sweetness (*β =* 0.29, *SE =* 0.054, *t =* 5.30, *p <* .001). Hunger also showed a small but significant negative association (*β =* −0.31, *SE =* 0.15, *t =* −2.06, *p* = .040). Familiarity and confidence did not significantly predict sweetness ratings. Overall, in the Salty-to-Sweet direction, expectancy context strongly shaped anticipatory sweetness, and sensory experience reversed these expectations most clearly in the Unexpected condition, producing a robust expectancy-violation contrast effect that differed qualitatively from the Sweet-to-Salty trajectory.

#### 3.3.3 Sensory reweighting: saltiness

Saltiness ratings (Fig. 3) showed pronounced anticipatory modulation across expectancy conditions, with the largest effects emerging prior to tasting. In the Expected condition, saltiness was low at both time points, declining slightly from *M =* 1.98 (*SD =* 1.35) before tasting to *M =* 1.52 (*SD =* 1.15) after tasting (Δ = 0.46). In the Neutral condition, saltiness decreased substantially from *M =* 3.70 (*SD =* 2.21) before tasting to *M =* 1.75 (*SD =* 1.37) after tasting (Δ = 1.95). The most pronounced decrease was observed in the Unexpected condition, where saltiness dropped sharply from *M =* 5.22 (*SD =* 1.78) before tasting to *M =* 2.00 (*SD =* 1.58) after tasting (Δ = 3.22). Thus, anticipatory saltiness was strongly elevated under Unexpected labeling but declined markedly following tasting. The mixed-effects model revealed no significant main effect of Time in the reference (Expected) condition (*β =* 0.22, *SE =* 0.22, *t =* 0.97, *p =* .331). However, this effect was strongly qualified by significant Condition × Time interactions. Relative to Expected, the before-after difference was significantly larger in the Neutral condition (*β =* 1.46, *SE =* 0.37, *t =* 3.92, *p <* .001) and dramatically larger in the Unexpected condition (*β =* 2.61, *SE =* 0.31, *t =* 8.36, *p <* .001), indicating a graded expectancy-sensitive re-evaluation. Follow-up contrasts confirmed that the before-after decrease was significant in the Neutral (Δ = 1.68, *SE =* 0.33, *p <* .001) and Unexpected (Δ = 2.82, *SE =* 0.25, *p <* .001) conditions, but not in the Expected condition (Δ = 0.22, *SE =* 0.23, p = .341). At the after-taste stage, most condition differences were eliminated; however, saltiness remained slightly higher in the Unexpected than in the Expected condition (Holm-adjusted *p* = .047), indicating near but not complete post-taste convergence. In contrast, all three conditions differed significantly at the before-taste stage (all *p*s < .001), with Unexpected showing the highest anticipatory saltiness, Neutral intermediate, and Expected the lowest. Among the covariates, perceived strength was a strong positive predictor of saltiness (*β =* 0.22, *SE =* 0.041, *t =* 5.40, *p <* .001). Familiarity, confidence, and hunger did not significantly predict saltiness ratings. Overall, in the Salty-to-Sweet direction, expectancy context strongly amplified anticipatory saltiness judgments, and tasting sharply reduced these differences, particularly under Neutral and Unexpected labelling, though a small residual difference remained between Expected and Unexpected conditions after tasting.

## 4 Discussion

The present study demonstrates that misleading linguistic labels systematically structure anticipatory flavour perception, and that the magnitude and symmetry of contrast effects depend on flavour trajectory. Across both Sweet-to-Salty and Salty-to-Sweet directions, expectancy manipulations exerted their strongest influence prior to tasting, with large pre-ingestive divergences that were subsequently attenuated or recalibrated following sensory input. However, the form of this recalibration differed across trajectories and judgment types. In the Sweet-to-Salty direction, expectancy violations produced coordinated hedonic and sensory contrast. Labelling savoury-dominant foods as sweet inflated anticipatory pleasantness and sweetness, which then collapsed sharply after tasting, accompanied by increased perceived saltiness. These effects were expressed as graded Condition × Time interactions, indicating dynamic within-participant re-evaluation rather than static between-condition differences. In the Salty-to-Sweet direction, sensory contrast effects were again robust, particularly for sweetness and saltiness, whereas hedonic contrast appeared less pronounced than in the Sweet-to-Salty direction. However, because no omnibus cross-trajectory model was fitted, this apparent asymmetry should be interpreted cautiously. Anticipatory ratings diverged strongly across conditions and often reversed following tasting, yet pleasantness showed a general pre-post decline without a graded interaction. Thus, while sensory recalibration was bidirectional, hedonic re-evaluation was more pronounced when sweet expectations were violated by savoury-dominant foods than when savoury expectations were violated by sweet food stimuli. Taken together, these findings indicate that linguistic labels primarily shape flavour perception by structuring pre-ingestive expectations, which are then updated in light of sensory evidence. Expectancy-driven contrast is therefore robust across flavour polarities, but its expression is dimension-specific and dynamically constrained by the unfolding sensory profile rather than reflecting a uniform response to mismatch.

### 4.1 Replication and broadening of the smoked salmon ice-cream effect

The present findings replicate the core smoked salmon ice-cream effect and broaden the paradigm beyond its original single-item format. Consistent with Yeomans et al. (2008), incongruent sweet labelling produced inflated anticipatory sweetness and pleasantness that were sharply reduced following tasting, accompanied by enhanced perception of saltiness. This confirms that expectancy-driven contrast emerges robustly under label-only conditions and does not depend on supportive visual ambiguity. Beyond replication, the current design demonstrates that the effect is not confined to a single highly distinctive stimulus. By generalising the paradigm across multiple foods and reversing flavour polarity, we show that contrast is a systematic consequence of expectancy violation rather than an idiosyncratic feature of smoked salmon ice-cream. The inclusion of both Sweet-to-Salty and Salty-to-Sweet trajectories further reveals that sensory recalibration is bidirectional, while also suggesting that the hedonic consequences of expectancy violation may differ by flavour direction. Because this comparison is based on separate trajectory-specific models, however, the directional asymmetry should be regarded as provisional rather than definitively established. Any apparent directional difference should also be interpreted in light of the fact that the two trajectories were instantiated by different foods, which may have differed in familiarity, baseline liking and perceived plausibility independent of expectancy violation. Importantly, the coordinated shifts in sweetness and saltiness across trajectories indicate that expectancy violations reorganise perceptual weighting across flavour dimensions, rather than merely reducing overall liking. The dissociation between hedonic evaluation and specific taste attributes reinforces the view that contrast effects reflect dynamic updating of sensory representations when predictions are disconfirmed. In this respect, the present study moves the smoked salmon paradigm from a single-item demonstration to a more general account of how misleading labels reshape flavour perception.

### 4.2 Isolating linguistic expectations from visual cues

A key methodological advance of the present study is the isolation of linguistic expectations from visual appearance. In the original smoked salmon ice-cream paradigm, expectations were shaped jointly by ambiguous visual presentation and verbal description. Here, pre-taste evaluations were elicited from written labels alone, allowing a more direct test of whether language itself is sufficient to generate structured flavour predictions. Across both flavour trajectories, labels produced strong anticipatory differences in sweetness, saltiness and pleasantness, demonstrating that verbal information alone can establish robust perceptual priors. This finding is consistent with a broader literature showing that food-extrinsic cues, including labels and descriptions, systematically shape anticipated and experienced flavour (Deliza and MacFie, 1996; Piqueras-Fiszman and Spence, 2015).

Importantly, these label-driven expectations were not merely cognitive inferences but perceptually consequential and dynamically updated. Large pre-taste divergences were consistently reduced, reversed or recalibrated following ingestion, indicating that language structures pre-ingestive simulation, which is then revised in light of sensory evidence. The fact that these effects emerged in the absence of supportive visual cues underscores the potency of verbal labels as prior-setting signals in flavour perception. Together with the replication findings, this strengthens the conclusion that expectancy-driven contrast does not depend on multisensory ambiguity but can arise from linguistic framing alone. It is also important to note that the neutral labels used here should not be interpreted as eliminating expectation altogether. A generic food label may still invite weak default inferences, even if it does not specify flavour direction. The Neutral condition therefore provides a low-information comparison point rather than a psychologically expectation-free baseline.

### 4.3 Apparent directional asymmetry and the dynamics of expectancy violation

In the Sweet-to-Salty direction, mislabelling a savoury product as *smoothie, mousse, ice-cream, gummy, yoghurt* generated strong sweet-fruity priors, closely mirroring the anticipatory profile documented in Experiment 3 of Yeomans et al. (2008), where the ice-cream label elicited high expected sweetness and pleasantness prior to tasting. In both cases, these priors were sharply disconfirmed, yielding pronounced hedonic decline and amplification of unexpected saltiness, consistent with contrast-based exaggeration of unexpected sensory attributes (Carlsmith and Aronson, 1963; Yeomans et al., 2008). By contrast, in the Salty-to-Sweet trajectory, although anticipatory divergence was equally strong, post-ingestive adjustment involved a partial reversal (sweetness increased, saltiness decreased under Unexpected labelling) rather than a simple exaggeration of the initially unexpected dimension.

Although expectancy violation produced robust contrast effects in both flavour trajectories, the pattern of recalibration appeared not to be fully symmetrical. Importantly, however, this impression may reflect not only differences in expectancy dynamics, but also item-level differences in baseline acceptability, familiarity and cultural plausibility. Some Salty-to-Sweet combinations may be perceived as unusual but still interpretable as edible, whereas some Sweet-to-Salty items may be more immediately aversive independent of the specific label manipulation. A further possibility is that the asymmetry partly reflects functional biases in the chemical senses themselves. Sweet taste is commonly associated with energy-rich foods and reward, whereas bitter and sour cues, and in some contexts other sharply disconfirming flavour notes, are more closely tied to rejection and defensive responding in feeding behaviour (Breslin, 2013). From this perspective, an abrupt shift from an expected sweet dessert-like profile to savoury or fermented notes may be especially disruptive because it violates not only a learned food schema but also a more basic evaluative bias favouring sweet over aversive or warning-like oral signals (Reed and Knaapila, 2010).

Another possible interpretation of this pattern is that violations of a dominant sweet schema (*e.g.*, ice-cream) may trigger more immediate affective rejection and sharper sensory contrast, both because the expected category is strongly learned and because abrupt deviation from a sweet prior may engage broader food-rejection tendencies. At the same time, an alternative explanation is that the two stimulus sets differed in their baseline plausibility and acceptability. For example, several Salty-to-Sweet items may be culturally unusual but not intrinsically aversive, whereas some Sweet-to-Salty items may be harder to assimilate even before tasting because their ingredient combinations are less familiar or less acceptable. The present design does not fully disentangle these possibilities. The former interpretation aligns with broader models of assimilation and contrast (Hovland et al., 1957; Wilson et al., 1989; Schifferstein, 2001), which emphasise that the magnitude and direction of expectancy effects depend on both discrepancy size and prior strength. Importantly, we do not claim that sweet expectations are inherently more powerful than savoury ones; rather, the present data indicate that the dynamics of recalibration depend on the direction of flavour unfolding. Expectancy violation is therefore not a unitary phenomenon but is constrained by the trajectory of sensory evidence as it accrues. This interpretation should nonetheless remain tentative until tested in a combined model that directly evaluates whether the relevant Condition × Time interaction differs significantly by trajectory. It is a key limitation that trajectory comparisons were based on separate models for Sweet-to-Salty and Salty-to-Sweet items. Establishing whether the observed differences reflect a statistically reliable asymmetry would require a combined omnibus model including Trajectory as a factor and testing the Trajectory × Condition × Time interaction directly.

### 4.4 Implications for predictive processing and flavour perception

The present findings are broadly consistent with predictive coding accounts of perception, according to which experience reflects the integration of top-down predictions with bottom-up sensory input, weighted by their relative precision (Clark, 2013; Summerfield and de Lange, 2014). Across both flavour trajectories, linguistic labels generated strong anticipatory priors that structured sweetness, saltiness and pleasantness before ingestion. These priors were then recalibrated once sensory evidence accrued, producing systematic Condition × Time interactions rather than static between-label differences. Descriptively, updating appeared trajectory-sensitive. In the Sweet-to-Salty direction, the labels appear to have induced a high-precision sweet–pleasant prior that was sharply disconfirmed on tasting, yielding pronounced prediction error, collapse of anticipated sweetness, amplification of saltiness and graded hedonic decline. This pattern can be interpreted within a predictive-processing framework, but the present design does not directly measure prediction error or prior precision. In the Salty-to-Sweet direction, anticipatory priors were again sharply differentiated by label, but post-ingestive updating involved partial reversals (*e.g.*, increases in sweetness and decreases in saltiness under Unexpected labelling), a pattern that is consistent with dynamic rebalancing of perceptual weights rather than uniform rejection. A further possibility is that prediction error magnitude was partly shaped by item-level priors grounded in prior cultural experience with particular food combinations rather than by label incongruence alone.

Across datasets, condition differences were largest before tasting and largely attenuated afterward, indicating that labels primarily structured pre-ingestive simulation and that sensory evidence subsequently reduced prior-driven disparities. From a predictive perspective, expectancy violations may be interpreted as involving triggering redistribution of perceptual weighting across dimensions: disconfirmed features are down-weighted, while diagnostically informative but initially underweighted attributes gain salience. The accompanying shifts in pleasantness may, within this framework, be interpreted as an affective correlate of prediction error (Wilson et al., 1989; Yeomans et al., 2008), rather than as an independent evaluative process. In sum, the data suggest that flavour contrast effects are compatible with general mechanisms of expectation updating (Piqueras-Fiszman and Spence, 2015), instantiated here in a trajectory-dependent manner. Within this interpretive framework, expectancy violation is therefore not merely a hedonic phenomenon, but a structured process of perceptual reorganisation unfolding over time as predictions are confronted with sensory input. At the same time, the present study does not directly estimate computational parameters such as prior precision, prediction error magnitude or updating weights. Formal tests of these mechanisms would require computational modelling or designs that manipulate these factors more directly.

### 4.5 Conclusion

The present study replicates the core smoked salmon ice-cream effect and broadens the paradigm by testing label-driven expectancy violation across multiple foods and across two flavour trajectories. By testing five Sweet-to-Salty and five Salty-to-Sweet foods, we show that strong expectancy violations reliably produce structured Label × Time interactions in both sensory and hedonic domains. Across trajectories, linguistic labels alone were sufficient to establish robust anticipatory priors for sweetness, saltiness and pleasantness. These priors were most evident before tasting and were systematically recalibrated once sensory evidence became available. In the Sweet-to-Salty direction, misleading sweet labels produced graded hedonic decline, collapse of anticipated sweetness and amplification of saltiness. In the Salty-to-Sweet direction, expectancy effects were equally strong at the anticipatory stage but manifested post-ingestively as cross-over or convergence patterns. These differences suggest a possible trajectory-sensitive component in flavour reweighting, but this interpretation remains provisional in the absence of a formal omnibus cross-trajectory test. Because the Sweet-to-Salty and Salty-to-Sweet trajectories were instantiated by different foods, future work will benefit from matching stimuli more tightly on baseline familiarity, acceptability and cultural plausibility in order to isolate expectancy-driven asymmetry more cleanly. A further design limitation is that the 4:4:2 allocation scheme gave fewer trials to the Neutral condition than to the Expected and Unexpected conditions. Although this was appropriate for prioritising the main expectancy contrast, future studies could use more balanced designs and directly assess default expectations in the neutral condition to determine how far generic labels themselves shape anticipatory judgments.

Condition differences were consistently largest prior to ingestion and largely attenuated afterward, underscoring the anticipatory locus of label-driven modulation. The findings therefore indicate that flavour contrast effects reflect dynamic updating of perceptual weighting across dimensions, rather than static shifts in liking or simple assimilation-contrast outcomes. From a food perception perspective, the results highlight the potency of linguistic labelling as a prior-setting cue and demonstrate that its effects are robust across diverse stimulus configurations. More broadly, they suggest that consumer experience is shaped as much by pre-ingestive simulation as by sensory input itself, with clear implications for product labelling and applied sensory research. At the same time, these implications should be interpreted within the limits of the present design, which used unusual stimuli, a single Mandarin-dominant cultural-linguistic sample and a label-only procedure without visual cues. The extent to which the same effects generalise to more typical foods, other populations and richer multisensory consumer contexts remains an important question for future research.

## Ethics statement

The study was conducted in accordance with the ethical standards of the institutional research ethics committee and with the principles of the Declaration of Helsinki. Ethical approval was obtained from the University of Auckland Human Participants Ethics Committee (Reference 25922; *Taste and Language: Linguistic modulation of gustatory processing*) prior to data collection. All participants provided written informed consent before taking part in the study and were free to withdraw at any time without penalty. Participants reported no food allergies, dietary restrictions, or health conditions that could affect taste perception. Data were collected and analysed anonymously.

## Declaration of competing interest

The authors declare that they have no known competing financial interests or personal relationships that could have appeared to influence the work reported in this paper.
