# Supplementary material for: Hedonic contrast under misleading labels: Bidirectional effects across sweet and salty foods
Source: Curr Res Food Sci. 2026 May 1;12:101420. doi: 10.1016/j.crfs.2026.101420 (PMC13156992; doi:10.1016/j.crfs.2026.101420)
Supplement: Multimedia component 1 [file mmc1.docx]

**Hedonic contrast under misleading labels:**

**Bidirectional effects across sweet and salty foods**

**Ethics statement**

The study was conducted in accordance with the ethical standards of the institutional research ethics committee and with the principles of the Declaration of Helsinki. Ethical approval was obtained from the University of Auckland Human Participants Ethics Committee (Reference 25922; *Taste and Language: Linguistic modulation of gustatory processing*) prior to data collection. All participants provided written informed consent before taking part in the study and were free to withdraw at any time without penalty. Participants reported no food allergies, dietary restrictions, or health conditions that could affect taste perception. Data were collected and analysed anonymously.
